# Comparison of reprogramming factor targets reveals both species-specific and conserved mechanisms in early iPSC reprogramming

**DOI:** 10.1186/s12864-018-5326-1

**Published:** 2018-12-22

**Authors:** Kai Fu, Constantinos Chronis, Abdenour Soufi, Giancarlo Bonora, Miguel Edwards, Stephen T. Smale, Kenneth S. Zaret, Kathrin Plath, Matteo Pellegrini

**Affiliations:** 1 Department of Molecular, Cellular and Developmental Biology, Bioinformatics Interdepartmental Program, University of California Los Angeles, Los Angeles, CA 90095 USA; 2 David Geffen School of Medicine, Department of Biological Chemistry, Eli and Edythe Broad Center of Regenerative Medicine and Stem Cell Research, Jonsson Comprehensive Cancer Center, Molecular Biology Institute, Bioinformatics Interdepartmental Program, University of California Los Angeles, Los Angeles, CA 90095 USA; 3 Institute for Regenerative Medicine and Epigenetics Program, Department of Cell and Developmental Biology, Smilow Center for Translational Research, University of Pennsylvania Perelman School of Medicine, Philadelphia, PA 19104 USA; 4 Department of Microbiology, Immunology and Molecular Genetics, University of California Los Angeles, Los Angeles, CA 90095 USA; 5 Present address: University of Edinburgh, MRC Centre for Regenerative Medicine, SCRM Building, Edinburgh Bioquarter, Edinburgh, EH16 4UU UK

**Keywords:** Reprogramming, iPS cells, Comparative epigenomics

## Abstract

**Background:**

Both human and mouse fibroblasts can be reprogrammed to pluripotency with Oct4, Sox2, Klf4, and c-Myc (OSKM) transcription factors. While both systems generate pluripotency, human reprogramming takes considerably longer than mouse.

**Results:**

To assess additional similarities and differences, we sought to compare the binding of the reprogramming factors between the two systems. In human fibroblasts, the OSK factors initially target many more closed chromatin sites compared to mouse. Despite this difference, the intra- and intergenic distribution of target sites, target genes, primary binding motifs, and combinatorial binding patterns between the reprogramming factors are largely shared. However, while many OSKM binding events in early mouse cell reprogramming occur in syntenic regions, only a limited number is conserved in human.

**Conclusions:**

Our findings suggest similar general effects of OSKM binding across these two species, even though the detailed regulatory networks have diverged significantly.

**Electronic supplementary material:**

The online version of this article (10.1186/s12864-018-5326-1) contains supplementary material, which is available to authorized users.

## Background

By expressing the transcription factors Oct4, Sox2, Klf4 and c-Myc (abbreviated as OSKM), differentiated cells can be reprogrammed into induced pluripotent stem cells (iPSCs) that have the ability to differentiate into any type of cell [1, 2]. iPSC technology holds great promise in regenerative medicine and for the modeling of diseases in a culture dish [3, 4]. However, there is still limited understanding of the essential mechanisms underlying reprogramming of somatic cells to iPSCs. Furthermore, there are marked differences in the reprogramming process for mouse and human cells, even though reprogramming can be accomplished by the same set of factors. Mouse cells reprogram within a week or two, whereas human cells may take up to a month and the efficiency of the conversion is typically lower in the human system [5, 6]. Moreover, while mouse cells can be reprogrammed efficiently with OSK alone, ectopic c-Myc expression is more critical in the human process [1, 2, 7]. To understand universal features of reprogramming across species, we characterized the differences and similarities in the regulatory networks that were manifested at the onset of reprogramming of human and mouse somatic cells.

An important approach towards understanding the reprogramming process is to systematically investigate the binding of reprogramming factors in the genome. By investigating OSKM binding at 48 h of reprogramming, previous studies have begun to elucidate the patterns and regulatory roles of OSKM in early reprogramming in the human and mouse systems [8–10]. Reprogramming typically is an inefficient process where only few cells in the culture dish induce the pluripotency program, yielding a highly heterogeneous cell population at the end of the process [11]. In previous studies, Koche et al. demonstrated that within the first few days of iPS reprogramming, dividing and nondividing cells exhibited highly similar transcriptional profiles [12]. Polo et al. showed that in the first three days of reprogramming, cells sorted for reprogramming markers and analyzed by transcriptomics clustered together [13]. These studies thus indicate a significant degree of homogeneity in cellular responses during the early time scale of reprogramming, enabling location studies of OSKM in the early reprogramming population. Moreover, for the 48-h time point in mouse, we used fetal bovine serum containing media, which results in iPSC colonies within 2–3 weeks. In these conditions, the timing of reprogramming is similar to that found in human experiments. The early human and mouse cells are thus expected to be in a similar stage of reprogramming. However, the final iPSC stage between human and mouse is significantly different: the human cells are reprogrammed to a primed stage while the mouse cells are reprogrammed to a naïve stage [14]. For those reasons, in this study we focused on the 48-h comparison instead of the iPSC stage of reprogramming.

In this study, we compared the initial OSKM binding events between human and mouse fibroblasts to shed light on both conserved and species-specific mechanisms of OSKM-mediated processes early in reprogramming. The mouse system used transgenic mouse embryonic fibroblasts, thus every cell expresses the four factors [10]. Similarly, in the ectopic expression of OSKM in human fibroblast, each of the factors were shown to be expressed in 97–99% of the cells [8]. Thus, it is unlikely that heterogeneity in expression will lead to differences between the mouse and human systems being studied. Moreover, by focusing on the binding events of OSKM early in reprogramming, we guaranteed minimal influence of the differences between human and mouse cell reprogramming that resulted in mouse iPSCs in the naïve pluripotent state and human iPSCs in the primed pluripotent state caused by the external culture conditions.

We first show that general features of OSKM binding events, such as inter- and intragenic distribution, target genes, primary binding motifs, and combinatorial binding patterns between the reprogramming factors, are largely similar between human and mouse. However, when we compared the locations of OSKM binding events, we found that only a small fraction of binding sites in syntenic regions were conserved between human and mouse at 48 h of reprogramming. This result indicates that the binding of the reprogramming factors is in large part distinct at the initial stage of the reprogramming process. We show that conserved binding events within syntenic regions often represent target sites that are also bound in the pluripotent end state and tend to occur in promoters and enhancers, suggesting that the engagement of pluripotency sites early in reprogramming is a conserved mechanism between mouse and human reprogramming. Lastly, we show that both motif usage and chromatin states contribute to the conservation of binding events in early human and mouse reprogramming.

## Results

### General features of OSKM binding events in early human and mouse reprogramming

In this study, we compare the binding of OSKM peaks in mouse and human at 48 h post transfection. This is accomplished by analyzing previously published datasets [8, 10]. We note that there are some differences in the mouse and human datasets that are due to the difference in overexpression methodology and the starting cell type. While the mouse data was generated by overexpressing the pluripotency factors using a polycistronic cassette (ensuring that each cell expresses all four factors at comparable levels), the human data was generated using individual lentiviral vectors, which leads to more variability in the combination and level of expression of the factors. However, as we show below, these differences do not have a significant impact on our conclusions.

We first addressed the effects of overexpression between polycistronic and individual based approaches. While the primary results presented in Chronis et al were based on a polycistronic cassette [10], in the same study we also collected binding data generated by individually overexpressing factors using pmX. We showed that in mouse, individual retroviral based expression of Oct4, Sox2 and Klf4 (OSK) have strong signals in the polycistronic derived OSK peaks, indicating that the OSK signal from the two systems are enriched in similar genomic loci (Additional file 1: Figure S1). In addition, the two protocols yielded significant number of overlapped peaks (Additional file 1: Figure S2). Moreover, we note that while the mouse experiments were carried out in embryonic fibroblasts, the human studies were done in fetal foreskin fibroblast. Since we did not have access to epigenomes from both embryonic fibroblasts and fetal foreskin fibroblasts in either human or mouse, we were not able to compare the potential differences between the two starting cell types. Nonetheless, we do have access to epigenomes for both human foreskin newborn and human lung fetal fibroblasts from Roadmap Epigenomics Project. To address the potential differences between different types of fibroblasts, we used DNaseI hypersensitive sites to represent the chromatin states and then compared their overlapping. Additional file 1: Figure S3 then shows that although there are differences, the two types of fibroblasts have a significantly overlapped number of DNaseI peaks, suggesting the overall similarities of chromatin states between those two types of fibroblasts.

Having shown that these two experimental strategies yield similar OSKM binding events, we chose to focus our analyses on the mouse polycistronic and human individual lentiviral cassette where all our ChIP-Seq was collected. To further enable their comparison, we generated OSKM peaks for both human and mouse cells reprogramming using the same analysis pipeline for mapping and peak calling, setting the peak calling q-value cutoff of 0.05 (see Methods).

**Fig. 1:**
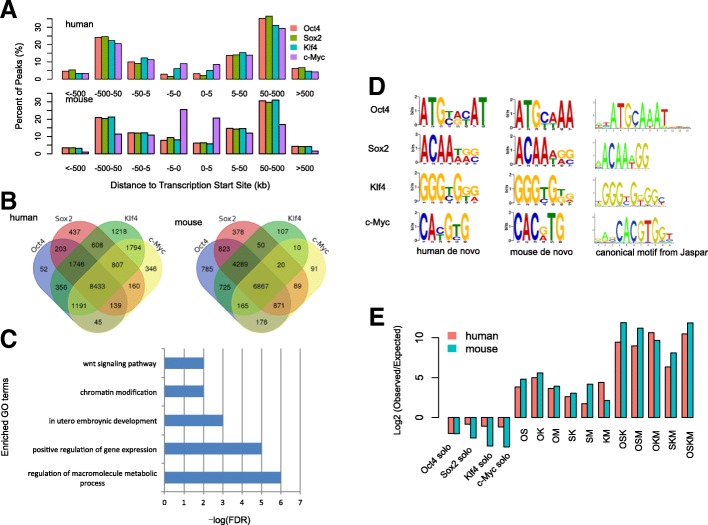
General feature comparison of OSKM ChIP-Seq peaks between human and mouse 48 h fibroblast reprogramming. **a** Positional distribution of OSKM peaks with respect to Transcription Start Sites (TSSs). The top panel shows the peaks in human while the bottom panel shows that in mouse. **b** Venn diagram of OSKM co-targeted genes in human (left panel) and in mouse (right panel). **c** Enriched gene ontology terms (biological process) of shared OSKM co-targeted genes between mouse and human. **d** De novo and canonical motifs of OSKM peaks. **e** Log2 ratio of observed combinatorial binding events versus expected

The human and mouse data sets generated a similar number of peaks for Oct4, while the early human reprogramming culture had about twice as many peaks for the other three factors compared to the mouse (Additional file 1: Figure S4). We then first asked whether OSKM peaks had a similar positional distribution with respect to transcriptional start sites (TSSs) in the two species (Fig. 1a). Specifically, we classified the distances between peaks and TSSs into different groups, i.e. 0 to 5 kb, 5 to 50 kb etc. As has shown before [8, 10], O, S and K in both human and mouse predominantly bind regions distal to TSS. However, in humans M tends to bind distally to the TSS whereas in mouse it tends to bind proximal to TSS regions. This result is consistent with previous finding that M has a different distribution between human and mouse [8, 10] (Fig. 1a).

We next compared the target orthologous genes for each factor between human and mouse reprogramming. Targets were defined as an orthologous gene whose TSS is closest to the peaks for each factor irrespective of binding distance. We then calculated the number of overlapping target genes among the four factors in the two species and found that a large fraction of orthologous genes was targeted by the four factors in both species (Fig. 1b). Furthermore, among the 8433 OSKM co-targeted genes in human and 6867 co-targeted genes in mouse, 3919 of 15,789 orthologous genes were shared significantly (*p*-value < 10^− 16^, hypergeometric test), indicating a significant number of OSKM co-targeted genes were conserved, even though there was still a large set of genes were not shared between the species. Gene ontology enrichment analyses showed those shared co-targeted genes were enriched in the biological processes of regulation of macromolecular metabolic process, regulation of transcription, in utero embryonic development and regulation of Wnt signaling pathway (Fig. 1c). This agrees with previous studies which showed that the Wnt signaling pathway modulated reprogramming efficiency when altered early in reprogramming [15].

We next carried out de novo motif discovery in each factor’s binding regions (see Methods). The DNA binding motifs we identified for each reprogramming factor was similar between human and mouse (Fig. 1d). We also observed minor motif differences in Oct4, which terminated with A/T AA in mouse but A/G C/T AT in human, as well as in c-Myc, which terminated with C G/A TG in mouse but C/T G T/C G in human. We further used STAMP, which is a web tool for exploring DNA-binding motif similarities, to compare the reprogramming factor motifs between human and mouse [16]. As a result, the motif similarity E-value was 1.14e^− 7^, 3.72e^− 8^, 3.44e^− 11^, 1.56e^− 8^ for O, S, K and M factor respectively. Thus, the motifs between human and mouse were significantly similar. Moreover, de novo motifs of the four factors were largely consistent with their canonical motifs (obtained from Jaspar database) [17], indicating DNA binding preferences of O, S, K, and M are largely conserved between human and mouse.

To further characterize OSKM binding, we identified all possible combinations of binding events. If summits of peaks from different reprogramming factors were within 100 bp of each other, we considered them to be “co-” binding events. If summits of peaks from one factor were at least 500 bp away from all other factors, we defined these as “solo” binding events. To gauge whether co-bound sites occurred more or less frequently than expected, we compared our counts to a synthetic null model for all possible combinations of factors (see Methods). We found that in both human and mouse, all co-binding events occurred more frequently than expected, whereas solo binding sites were observed less frequently than expected (Fig. 1e). OSKM, OSK, OSM, OKM and SKM co-binding events were the most prevalent combinations in both human and mouse. Moreover, solo binding sites were more likely in human than in mouse and nearly all co-binding events (except KM and OKM) were more prevalent in mouse. Regardless of the differences, these results indicate that O, S, K, and M tend to bind together with similar combinatorial patterns in both human and mouse, suggesting that the factors often co-bind to exert their actions. Overall, we conclude that the general properties of O, S, K and M are similar, although there are some observable differences.

### Comparison of OSKM binding to the chromatin state of starting cells

**Fig. 2:**
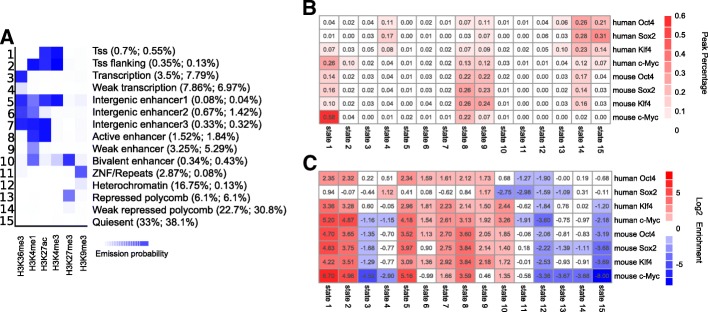
OSKM peaks target of the chromatin states in starting cells. **a** Chromatin state model for concatenated human and mouse fibroblast cells based on five histone marks. The value in the heatmap represents the enrichment of that histone mark in that learned chromatin state. The values in the bracket represent the genomic percentage (human then followed by mouse) occupied by that chromatin state. **b** Heatmap for percentages of OSKM peaks in each chromatin states from **a**. **c** Heatmap for log2 enrichments between OSKM peaks percentages and chromatin state genomic percentages

Next, we sought to compare the chromatin state in the starting cells for OSKM binding sites at 48 h between human and mouse. This enabled us to see how OSKM interacted with the initial chromatin states in fibroblasts. We analyzed H3K4me1, H3K4me3, H3K27me3, H3K27ac and H3K36me3 histone marks of human fibroblasts from the Roadmap Epigenome Project [18] and of mouse fibroblasts [10] to build a 15 chromatin state model using ChromHMM [19] with a concatenated human-mouse genome (see Methods). Based on the combinatorial probability of the five histone marks, we classified the mouse and human genomes into chromatin states such as active promoter and active enhancer. We chose a model with 15 chromatin states because these had a clearly distinct combination of histone marks and functional annotations based on prior expectations. The genomes of both human and mouse were segmented into non-overlapping 200 bp regions, and each bin associated with a specific chromatin state. Figure 2a shows the emission probabilities (signal enrichments) of each histone mark as well as the fractions of the genome (numbers in the brackets, human followed by mouse) that each chromatin state occupies in human and mouse fibroblasts. We noted primary differences between human and mouse chromatin states including the frequency of the two H3K9me3-containing chromatin states, weak repressed polycomb and quiescent chromatin state, where human fibroblasts had significantly more genomic regions annotated as ZNF/repeats and heterochromatin and less genomic regions annotated as the latter two states.

By intersecting OSKM peaks with chromatin states, we calculated the percentage of peaks within chromatin states in both human and mouse (Fig. 2b). As a result, about 40~50% of human O, S, and K peaks, and 20% of human M peaks were within low signal regions (states 14 and 15). This chromatin analyses agrees with a direct assessment of the individual histone modification states targeted by OSKM, which showed that O, S, and K predominantly target unmarked chromatin sites [8]. By contrast, in mouse, the percentage of low signal regions targeted decreased to 20% for O, S, and K peaks and 2% for M peaks. In addition, about 40~50% of mouse O, S, and K peaks and 30% of M peaks were within enhancers, consistent with the finding that mouse OSK efficiently target enhancers active in fibroblasts early in mouse reprogramming [10]. However, for human O, S and K peaks, this number dropped to about 10%~ 25% and M peaks showed a similar number of 30%. After correction for the genome percentage annotated as different chromatin states, the human peaks were still more enriched in low signal regions and less enriched in enhancer regions (Fig. 2c). Those results reveal a distinct distribution of OSKM in chromatin states of low signals and enhancers between human and mouse.

### OSKM binding events show limited conservation between human and mouse

**Fig. 3:**
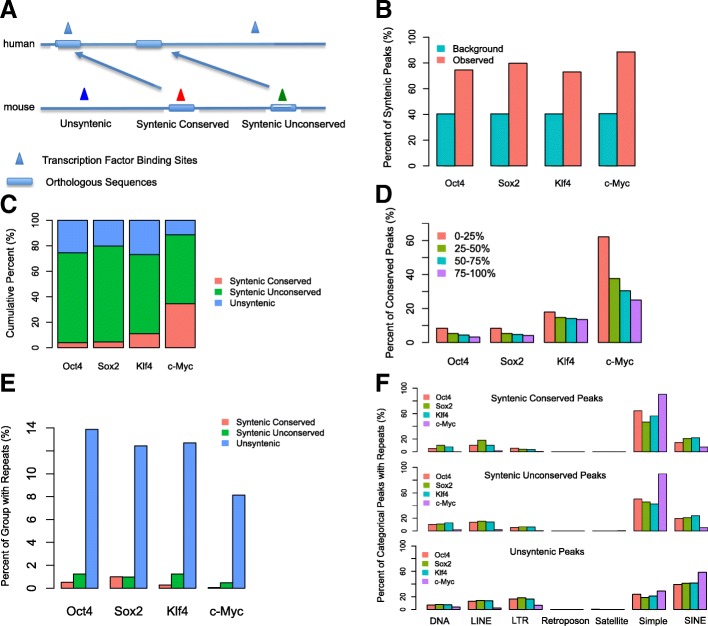
Map OSKM binding between human and mouse. **a** Schematic illustration of the three different groups of peaks, i.e. Syntenic Conserved (SC) binding group, Syntenic Unconserved (SU) binding group and UNsyntenic (UN) binding group. **b** Percentage of mouse OSKM peaks that can be mapped to human. The background is calculated by the simulation of peaks that have the same size and same number as the real peaks, and are allowed to map anywhere on the genome. **c** Fractional constitutions of SC, SU and UN peaks for each factor. **d** Percentage of SC binding events with respect to all syntenic binding events. For each factor, syntenic peaks are classified into four groups based on their peak enrichments of -log10(q-value). 0–25% are the top 25% of peaks while 75–100 are the bottom 25% of peaks. **e** Percentage of the three groups of peaks that contain repeat sequences. **f** Percentages of mouse peaks that contain specific type of mouse repeat sequences. Seven major types of repeat, i.e. DNA (DNA transposon elements), LINE (Long interspersed nuclear elements), LTR (Long terminal repeats), Retroposon (Transposons via RNA intermediates), Satellite (Satellite DNA which belongs to tandem repeats), Simple (Simple repeats) and SINE (Short interspersed nuclear elements) are calculated

To further compare OSKM occupancy in early mouse and human cell reprogramming, we mapped mouse peaks to the human genome based on synteny (see Methods). Mouse peaks were classified into three groups based on sequence conservation and binding conservation. Figure 3a shows a schematic illustration of the definition of the three groups: syntenic conserved peaks, syntenic unconserved peaks, and unsyntenic peaks. Syntenic conserved (SC) peaks had orthologous DNA sequences as well as binding events in both organisms. Syntenic unconserved (SU) peaks only had orthologous DNA sequences but no binding event detected in human. Unsyntenic (UN) peaks did not have orthologous DNA sequences between organisms and therefore could not be mapped between human and mouse.

We found that about 74, 80, 73 and 89% of mouse O, S, K, and M peaks, respectively, were syntenic with human, while the background ratio for the entire genome was about 40% (Fig. 3b), indicating that elements bound by OSKM show much higher sequence conservation rates than the rest of the genome, consistent with OSKM bind to cis-regulatory events such as enhancers and promoters. However, for each reprogramming factor, we found that syntenic conserved peaks only represented a small fraction of peaks (Fig. 3c). Specifically, 4, 4.5, 10.9 and 34.4% of mouse O, S, K, and M peaks, respectively, were syntenic conserved. O, S, and K, which mostly bind to enhancer regions in mouse (Fig. 2b) [10], had a lower fraction of conserved peaks compared to M, which mostly binds to promoter regions in early mouse cell reprogramming (Fig. 2b) [10]. We then asked whether the limited degree of conservation between mouse and human binding events could be solely explained by random background binding events between human and mouse. To address this we simulated both human and mouse background peaks (same number and length with the observed ones), then calculated the conservation rate and repeated the simulation 1000 times. The simulation result showed a conservation rate for OSKM background peaks of approximately 1%, implying that although the fraction of conserved binding was relatively small, conserved binding events still occurred at a higher rate than expected by chance. Lastly, we also mapped mouse pMX peaks (individual retroviral based system) to human peaks. Consistent with the comparison between polycistronic peaks in mouse and lentiviral peaks in human, our result showed that there was a limited fraction of syntenic conserved peaks for Oct4, Sox2 and Klf4 (Additional file 1: Figure S5). This result also indicates that the divergence of binding between human and mouse is not affected by using different overexpression systems.

In a previous study, Cheng et al. showed that the degree of binding conservation varied markedly, from several percent to about 60%, between human and mouse among different transcription factors (TFs) [20]. In addition, promoter bound TF binding sites showed higher conservation rates than enhancer sites. Moreover, this trend held after adjusting the sequence conservation differences between promoters and enhancers, indicating that the TF binding sites in promoter regions are indeed more conserved than those in enhancer regions [20]. In another study, Schmidt et al. reported a 10 to 22% binding conservation rate between two of five mammals for liver-specific transcription factors [21]. In early reprogramming, we observed a low conservation rate for O, S, and K and a medium conservation rate for M (Fig. 3c), indicating the significance of binding divergence in early reprogramming system between human and mouse fibroblasts.

We next investigated whether peak binding strength (based on peak calling q-values) had an impact on conservation. We classified all mouse peaks into four groups based on their -log10 q-values (Fig. 3d). For each reprogramming factor, we observed a clear trend where the strongest peaks (top 25%) had a higher percentage of syntenic conserved binding events compared to other three groups. This result suggests peak binding strength indeed is positively correlated with peak conservation rates and stronger peaks tend to be more conserved.

By analyzing the presence of repeat sequences within the three groups of peaks (see Methods) (SC, SU, and UN), we found that the unsyntenic peaks had a much higher percentage of repeat sequences compared to the other two groups for all factors. In addition, except for Sox2, syntenic conserved binding sites contained the fewest repeats (Fig. 3e). Moreover, compared to peaks in syntenic regions, peaks in unsyntenic regions were more often associated with long terminal repeats (LTR) and short interspersed nuclear elements (SINE) and less often with simple repeats in the mouse genome (Fig. 3f). These results are consistent with previous findings which showed that transposable elements are enriched in species-specific sequences and have rewired the transcriptional network during evolution [22, 23].

The analyses described above were carried out by mapping mouse OSKM peaks to the human genome, but we also performed the inverse analysis by mapping human OSKM peaks to the mouse genome (Additional file 1: Figure S6a). Approximately 60% of human peaks occurred in genomic regions syntenic with the mouse. The lower syntenic rate of human peaks mapping to the mouse genome compared with mouse peaks mapping to the human genome correlated with a higher proportion of repeats in human peak sequences (Additional file 1: Figure S7). Among human OSKM peaks in syntenic regions, those also found in the mouse (syntenic conserved) constituted a small proportion as seen in the reverse mapping of mouse OSKM peaks to the human data (Additional file 1: Figure S6b). Interestingly, syntenic and unsyntenic human OSKM peaks showed a more similar distribution of certain types of repeats compared to mouse peaks (Fig. 3f, Additional file 1: Figure S8).

We also investigated how human syntenic peaks and all peaks of mouse were distributed relative to each other. We first calculated the distances between human syntenic peak summits and mouse peak summits. We then categorized the distances into several groups of genomic ranges, i.e. within 200 bp, 400 bp, 600 bp, 800 bp etc. Lastly, to compare the observed distance distribution with simulated background, we calculated the background distance distribution, where the mouse peaks were shuffled and the human syntenic peaks were kept fixed. The result suggests that observed human syntenic peaks are indeed closer to observed mouse peaks than expected by chance (Additional file 1: Figure S9). Moreover, there was a clear trend showing that the log2 ratio between observed and simulated peaks declined with increased distance. Among the four factors, c-Myc showed the most dramatic trend. This is consistent with the fact that c-Myc is the most conserved factor compared to the other three.

### Syntenic conserved peaks are associated with different genomic features compared with unconserved peaks

**Fig. 4:**
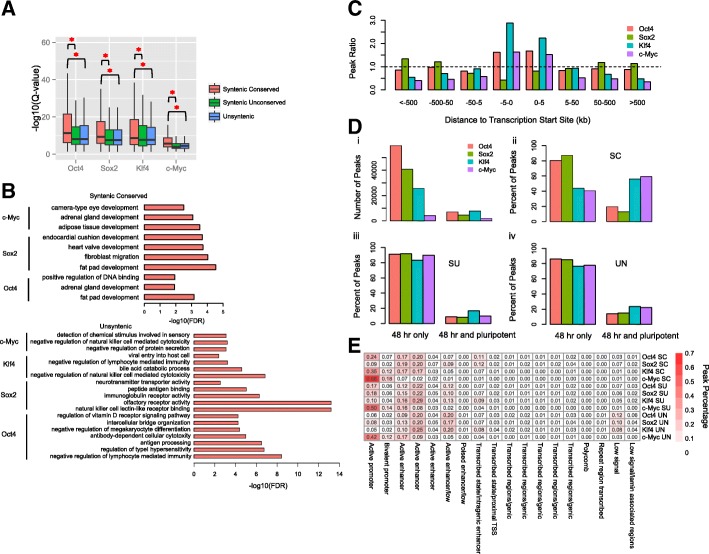
Comparisons of syntenic conserved peaks with syntenic unconserved peaks and unsyntenic peaks. **a** Box plot of peak calling q-values for the SC, SU and UN groups of peaks. **b** Enriched gene ontology terms for SC, SU and UN groups of peaks. **c** Fold enrichment of positional distribution between SC peaks and all peaks around Transcription Start Sites. **d** Percentage of SC, SU and UN peaks with consecutive bindings. 48 h only represents the peaks that only bound in 48 h of reprogramming, while 48 h and pluripotent represents the peaks that are also bound in the reprogramming final stage. i represents the number of the two group of peaks. ii-iv represents the percentage of SC, SU and UN peaks that are either 48 h only bound or 48 h and pluripotent bound. **e** Heatmap for percentages of mouse SC, SU and UN peaks in the mouse 18 chromatin states

Since we observed that only a small fraction of syntenic peaks had conserved binding early in reprogramming in human and mouse cells, we sought to identify properties that distinguish conserved peaks from the others. We observed that syntenic conserved peaks had significantly higher ChIP enrichment (−log10 q-value) than the other two groups (Fig. 4a), indicating the SC peaks tend to be bound more strongly. We then used the GREAT tool [24] to perform gene ontology enrichment analysis for the mouse SC, SU, and UN peaks, with all peaks as background (Fig. 4b). For SC peaks of OSM, we found their target genes were enriched for fat pad, adipose tissue, and adrenal gland development. Surprisingly, for SU peaks no enriched gene ontology terms for any of the four factors were detected. UN peaks of OSKM were strongly enriched in immunity-related gene ontologies. These results suggest that the target genes of the three groups of peaks might be associated with distinct functions. When comparing the genomic locations of mouse SC peaks to all peaks with respect to the distance to the TSSs, we found that SC O, K, and M peaks more often occurred within the proximal TSS regions, while Sox2 was slightly more often within the distal TSS regions (Fig. 4c).

We also compared binding of mouse OSKM at 48 h with that in the pluripotent state, to define those mouse OSKM binding events that were bound both early in reprogramming and in the pluripotent state (based on mouse embryonic stem cell ChIP-seq data) versus those that only occur at 48 h but not in pluripotent cells (Fig. 4d,i) [10]. In our previous study, we described that many of these persistent binding events for OSKM were enriched in promoters and OSK were also highly enriched in pluripotency enhancers [10]. We calculated the percentage of SC peaks that were bound only early in reprogramming or persist throughout reprogramming. We found that compared with SU and UN peaks, mouse SC peaks of OKM at 48 h had a higher fraction of persistent binding events (Fig. 4d,ii-iv). Specifically, for Oct4, the percent of persistent bound events was 20, 9 and 14 for SC, SU and UN respectively. For Klf4, this percent was 56, 17 and 23, and for c-Myc, this percent was 59, 10 and 22. This result indicates that conserved binding events, especially for K and M, tend to be maintained during reprogramming and are therefore likely to be more functionally important than unconserved ones.

We next asked whether SC, SU and UN peaks had distinct patterns of chromatin states in mouse at 48 h. A mouse 18 chromatin state model was generated with nine histone marks and described in our previous paper (Additional file 1: Figure S10) [10]. We therefore calculated the percentage of peaks within each chromatin state (Fig. 4e). As a result, we found that SC peaks preferentially tended to occur within certain chromatin states compared to SU and UN peaks. Specifically, SC peaks of O, K and M had higher percentages within active promoters, bivalent promoters and certain groups of enhancers. By contrast, UN peaks of O, S and K had higher percentages within low signal regions. Those results indicate that different groups of peaks are likely to associate with different chromatin states.

### Using transitions of regulatory motifs and chromatin states as predictors of conserved binding

**Fig. 5:**
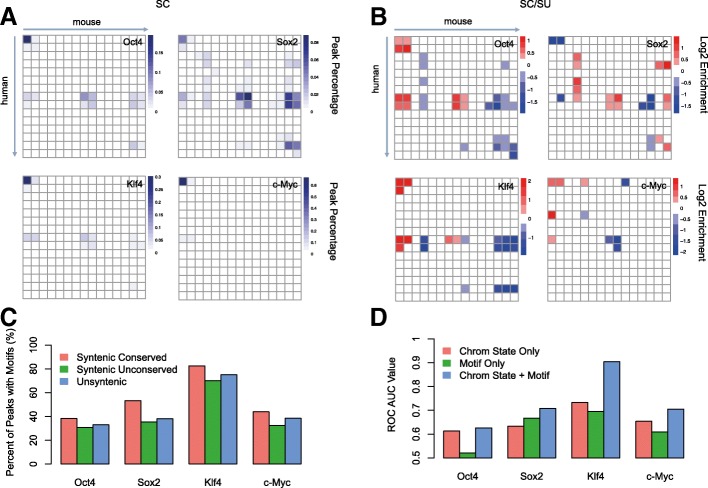
Chromatin state transitions, motif usages and their contributions in the maintaining of syntenic conserved peaks. **a** Chromatin state transitions of syntenic conserved peaks between human and mouse. The top left is state 1 of human to state 1 of mouse. The value in the heatmap represents the fraction of the number of syntenic conserved peaks in that square divided by the total number of all syntenic conserved peaks. **b** Chromatin state transitions of the log2 ratio between syntenic conserved peaks versus syntenic unconserved peaks. The value in the heatmap represents the log2 ratio between the fraction of syntenic conserved peaks and the fraction of syntenic unconserved peaks in that square. **c** Percentage of SC, SU and UN peaks that have canonical motifs. **d** ROC AUC of a classifier to predict syntenic based on motif occurrences and chromatin state transitions

To further investigate the chromatin states of syntenic peaks, we performed another comparison from a human-mouse transition perspective. We assigned each syntenic peak to the chromatin state in the concatenated human and mouse genome (Fig. 2a) and compared the chromatin assignment of each SC peak between mouse and human (see Methods) (Fig. 5a). The color in the heatmap reflects the percentage of SC peaks within that transition in chromatin state between the mouse and human syntenic genome. For example, the top left square in the heatmap is the transition from human TSS regions (state 1) to mouse TSS regions (state 1) and the bottom right is the transition from human quiescent regions (state 15) to mouse quiescent regions (state 15) (i.e. no changes in chromatin state), and any deviation from the diagonal represents a change in chromatin state. For SC peaks of O, S, and K, the most frequent transitions corresponded to human promoter to mouse promoter, human enhancer to mouse promoter, human enhancer to mouse enhancer, and human enhancer to mouse quiescent regions. By contrast, the majority of frequent transitions for c-Myc involved promoter to promoter states. We also asked whether the chromatin state transition patterns were different for unconserved peaks. When comparing the transition profiles between SC and SU peaks (Fig. 5b), we found an enrichment in human promoter to mouse promoter, human enhancer to mouse promoter and human enhancer to mouse enhancer transitions, indicating that SC peaks are more often associated with certain regulatory sites in both species than SU peaks.

Another factor that may help maintain the conservation of peaks is the occurrence of binding motifs. Although we observed that SC peaks were preferentially found within promoters and enhancers, it was not clear whether motifs help maintain the conservation of peaks between mouse and human. To shed light on this question, we computed the motif frequency in each group of peaks (see Methods) (Fig. 5c). We reasoned that if the conservation of peaks was strongly influenced by the presence of binding motifs between mouse and human, then SC peaks should have a different fraction of motifs compared to the other two groups. For Sox2, 53% of SC binding events had identifiable motifs within their peaks, compared to approximately 35% of SU and UN. However, for the other three factors, SC peaks contained more motifs but the differences among the three types of peaks were smaller, indicating the limited impact of sequence motifs in the determination of binding conservation.

To quantitatively assess the extent to which SC peaks are determined by motifs or chromatin states, we built a naïve Bayesian classifier to evaluate the prediction power for classifying syntenic peaks into the SC and SU groups (see Methods). This model was trained using different sources of information: motif only, chromatin state only, and the two combined. Area under the curve (AUC) values of receiver operator curves (ROC) were used to estimate the prediction power (Fig. 5d and Additional file 1: Figure S11). We found that except for Sox2, the chromatin state only model outperformed the motif only model. Moreover, when combining information from both motif and chromatin states, the AUC for O, S, K, and M were 0.63, 0.71, 0.90, and 0.71 respectively. Klf4 showed a strikingly high prediction power due to its strong motif preference in syntenic regions between human and mouse and its strong chromatin state preference for specific chromatin state transitions. Although the models for O, S, and M only predicted a fraction of conserved sites, these results demonstrate that conserved peaks are indeed associated with syntenic regions that contain strong motif sequences and preferred chromatin state transitions between mouse and human.

## Discussion

In this study, we systematically compared binding patterns of the four reprogramming factors OSKM between human and mouse at an early time point of reprogramming to the iPSC state. When analyzed in each genome separately, OSKM binding sites in human and mouse shared similar features: OSK tend to bind distal TSS regions, OSKM tend to target similar genes, have similar DNA binding motifs, and show similar combinatorial binding patterns among the reprogramming factors. This suggests that molecular properties of these factors are conserved between human and mouse. However, differences emerged when we investigated the chromatin state of target sites: OSKM targeted far more closed (low signal state) chromatin states in human cells than in mouse. Importantly, when we compared the binding sites across syntenic regions, we found that there was only a small percentage of sites that were bound in both genomes (i.e. syntenic conserved, SC). Altogether, our results suggest that the initial OSKM binding sites are largely distinct in these two species, even though the phenotypic consequences of these interactions ultimately lead to similar cell types.

We also observed that most early binding events do not persist in the later stages of iPSCs reprogramming [10]. However, we found that binding events that were conserved between mouse and human tended to persist more often throughout the reprogramming process compared to unconserved sites. Conserved binding sites also tended to have a higher proportion of conserved cis-regulatory elements associated with each factor. We also showed that binding sites were more likely to be conserved if the mouse and human chromatin states were similar and the motifs were conserved.

We recognize that there are certain limitations to our analysis. One is that human and mouse reprogramming was performed using slightly different experimental protocols. An inducible polycistronic cassette including all four reprogramming factors was used in mouse fibroblasts, ensuring homogeneous expression and stoichiometry across the cell population at 48 h; whereas four separate lentiviral constructs were used in human, each expressing one factor. However, as we have shown by comparing mouse polycistronic to individual cassettes, these different overexpression methods lead to very similar binding peaks. Also, it is possible that at 48 h, human and mouse cells might not be in the same reprogramming stages due to their different reprogramming kinetics. However, the time point we used corresponds to early events in the time series of both species, and should, therefore, identify the first interactions of these factors with chromatin. Moreover, we compared mouse embryonic fibroblasts and human fetal foreskin fibroblasts as starting cells of reprogramming. However, we believe that the epigenome changes from embryonic to fetal stages of fibroblasts are unlikely to have a dramatic effect on OSKM binding patterns. As a result, our conclusions drawn from the comparison of these two species should not be significantly affected by the differences in the experimental details of the human and mouse systems.

In conclusion, we have shown that while some general properties of OSKM binding are conserved between mouse and human, the specific genomic locations of transcription factor binding sites are vastly reorganized. A subset of the binding events are syntenic between the two species and this study has allowed us to identify these. We do not know if they represent key events that are distinct from the large fraction of other binding sites that are not conserved. However, several lines of evidence that we have presented, such as the fact that these sites tend to persist throughout the reprogramming process, do suggest that these may play a more significant role in reprogramming than the typical unconserved site. Nonetheless, the overall picture that emerges is that the OSKM regulatory networks have significantly diverged between the two species, and while the general properties of these networks are similar, the specific binding sites are generally distinct. This observation may suggest that reprogramming to pluripotency may be driven by global regulatory changes in cells that do not depend critically on a small set of specific interactions.

## Conclusions

By systematically compare the binding of the reprogramming factors, we are able to shed light on both conserved and species-specific mechanisms of OSKM-mediated processes early in reprogramming. In brief, there are mainly four significantly novel findings in this study. First of all, we found that general properties of reprogramming factors are largely shared between human and mouse in early iPSC stage. Secondly, the reprogramming factors initially target distinct chromatin states and induce different expression changes between human and mouse. Thirdly, only a small fraction of reprogramming binding events are conserved in early reprogramming. Lastly, the conserved binding events tend to represent the targets of the reprogramming factors in the pluripotent end state and could be partially predicted based on the presence of DNA motifs and chromatin states. Altogether, these findings suggest a similar mechanism in molecular properties perspective while the actual regulatory network of the pluripotency factors has been diverged significantly between the two species.

## Methods

### Cell culture and reprogramming

In the human reprogramming system, BJ fibroblasts were purchased from ATCC (CRL-2522) at passage 6 and cultured in the ATCC-formulated Eagle’s Minimum Essential Medium supplemented with 10% fetal bovine serum at 37 C and 5% CO2. The human H1-ES line [25] were purchased from ATCC and maintained as described [26]. More information about experimental details can be found in the supplementary documents of Soufi et al. 2012 [8]. In the mouse reprogramming system, the mouse embryonic fibroblasts were obtained from day 13.5 embryos of timed mouse pregnancies. The mouse embryos were obtained from Laboratory of K. Plath, where a cross between tetO OSKM/tetO OSKM and R26-M2rtTA/M2rtTA mice. The same mouse embryos were also used in Chronis et al. In addition, mouse embryonic fibroblasts carrying a polycistronic, dox-inducible OSKM cassette in the Col1A locus and a heterozygous M2rtTA allele in the R26 locus, were grown in standard mouse ESC media containing knockout-DMEM, 15% fetal bovine serum, recombinant leukemia inhibitory factor (Lif), b-mercaptoethanol, 1x penicillin/streptomycin, L-glutamine, and non-essential amino acids. Repogramming was induced by the addition of 2μg/ml doxycycline. We generated mouse iPS cell lines as described [27, 28]. Briefly, BJ cells at passage 10 were infected with lentiviruses encoding for dox-inducible Oct4, Sox2, Klf4, and c-Myc, along with lentiviruses expressing rtTA2M2 in the presence of 4.5 mg/ml polybrene. Additional experimental details can be found in the supplementary documents of Chronis et al. 2017 [10].

### Mapping and peak calling

The human OSKM ChIP-Seq datasets were downloaded from GEO with accession number of GSE36570, while mouse OSKM ChIP-Seq datasets were downloaded from GEO with accession number of GSE90895. Bowtie (version 0.12.8 and default parameters) was used to map ChIP-Seq reads of both human and mouse to their respective genomes. Only uniquely mapped reads were kept for further analysis [29]. MACS2 2.1.0 was used to identify ChIP-Seq peaks with a q-value cutoff of 0.05 (other parameters are in default) [30]. The human genome version is hg19 and the mouse genome version is mm9.

### Motif finding and motif occurrences within peaks

MEME-ChIP was used to perform de novo motif finding for OSKM binding peaks [31]. To identify the strongest motifs, the identified summits of peaks were ranked based on their enrichments and the top 10,000 summits, along with their surrounding 200 bp, were used as the input regions. The enriched motifs were identified using the DREME algorithm in the MEME-ChIP software. Starting with the most significant motif for each factor, we then used the Position Weight Matrix of this motif to scan for peaks, and determined the peaks associated with this motif using a *p*-value cutoff of 0.001.

### Combinatorial binding and solo binding

To identify combinatorial binding regions where multiple factors bind, peaks were merged if their summits were within 100 bp of each other. Then these different combinations of binding sites were broken down into their different combinations of factors. To identify solo binding regions where only one factor bound, we required that its summit be at least 500 bp away from all other factors. Note that this method is more stringent than that used by Soufi et al. [8]; the latter considered solo binding events as simply not falling within 100 bp of the peak center. Here, to estimate the background rates of combinatorial binding, the peaks of OSKM were first randomly shuffled in the genome (using the bedtools shuffle function) [32]. Secondly, the expected number of combinatorial binding events was re-calculated based on these shuffled peaks. Lastly, we compared the number of observed binding events versus the number of expected binding events for all possible combinations of factors.

### Mapping sequences between human and mouse

To map OSKM binding sites between human and mouse, the liftOver algorithm from the UCSC Genome Browser was used with a cutoff of 0.5. The LiftOver algorithm uses an alignment chain file to map genomic coordinates between different versions of assemblies, or different species. The algorithm searches for regions where the input sequences are in the same block with the converted assemblies or species. The cutoff of 0.5 requires that the mapped sequences share at least half of exactly same DNA sequences with the converted species. This cutoff is consistent with modENCODE project paper which compares transcription factor binding sites between human and mouse [20]. To confirm the reliability of our results, we also used another method named bnMapper and got very similar results [33].

### Peaks associated with repeat sequences

Repeat sequences were downloaded from the RepeatMasker database. We extracted the genomic coordinates for the major repeat families including DNA (DNA transposon elements), LINE (Long interspersed nuclear elements), LTR (Long terminal repeats), Retroposon (Transposons via RNA intermediates), Satellite (Satellite DNA which belongs to tandem repeats), Simple (Simple repeats) and SINE (Short interspersed nuclear elements). A peak was considered to be associated with a repeat sequence if the genomic coordinate of this repeat was within this peak.

### Chromatin states for concatenated human and mouse genomes

For mouse histone marks, we used the datasets for mouse fibroblast cells from our previously published paper [10]. For human histone marks, we used the datasets of IMR90 fibroblast cell line downloaded from RoadMap Epigenomics Project [18]. To learn the joint chromatin state for human and mouse, a pseudo chromosome size table was constructed by concatenating human and mouse genomes. Then the model was trained with the human fibroblast and mouse embryonic fibroblast histone data, producing a common set of emission probabilities. We then generated a 15 chromatin state model based on the combinatorial patterns of five histone marks, i.e., H3K4me1, H3K4me3, H3K27me3, H3K27ac and H3K36me3.

### Chromatin state transitions between human and mouse

Each syntenic conserved peak in mouse and its corresponding orthologous peak in human was assigned a chromatin state as described above. We then calculated the number of peaks within each possible chromatin state transition. This leads to the generation of a 15 X 15 chromatin state transition matrix. For example, the top left of the matrix represents the fraction of syntenic peaks with state 1 of human and state 1 of mouse. We also performed the same calculation for syntenic unconserved peaks between human and mouse. To compare to relative enrichment of chromatin state transitions, the log2 ratio between the syntenic conserved and syntenic unconserved matrices was calculated.

### Classification model

We built a Naïve Bayes model to classify syntenic peaks into a syntenic conserved and syntenic unconserved group, based on their chromatin state transition (see above) and motif occurrences transitions. The motif occurrence transition matrix was a 2 X 2 matrix that represents the frequency of motif occurrences for syntenic peaks between human and mouse. Log odds ratios were then calculated between syntenic conserved group and syntenic unconserved groups for both chromatin state transition and motif occurrence transition matrices. As a result, each peak was assigned two values: one was the chromatin state transition log odds ratio matrix to represent the chromatin state model, and another was the motif occurrences transition log odds ratio matrix to represent the motif model. The two values were added to represent both the chromatin state and motif occurrence model. Syntenic peaks were then ranked based on log odds ratio values from either the chromatin state transition matrix or motif occurrences transition matrix, or their sum. A syntenic conserved peak was labeled as 1 and a syntenic unconserved peak is labeled as 0. Lastly, the Area under the curve (AUC) values of the receiver operator curves (ROC) were calculated to represent the model performance for classifying syntenic peaks into 1 or 0 given the chromatin state transitions or motif occurrences transitions.

## Additional files

Additional file 1: Additional analyses including further supporting materials for the major findings in the manuscript. (PDF 3330 kb)

## Abbreviations

AUC: Area under the curve
ChIP-Seq: Chromatin Immunoprecipitation followd by Sequencing
iPSCs: Induced pluripotent stem cells
LTR: Long terminal repeats
OSKM: Oct4, Sox2, Klf4 and c-Myc
ROC: Receiver operator curves
SC: Syntenic conserved
SINE: Short interspersed nuclear elements
SU: Syntenic unconserved
TF: Transcription factor
UN: Unsyntenic
