# Supplementary material for: Comparison of reprogramming factor targets reveals both species-specific and conserved mechanisms in early iPSC reprogramming
Source: BMC Genomics. 2018 Dec 22;19:956. doi: 10.1186/s12864-018-5326-1 (PMC6303873; doi:10.1186/s12864-018-5326-1)
Supplement: Supplementary file 1 — Additional analyses including further supporting materials for the major findings in the manuscript. (PDF 3330 kb) [file 12864_2018_5326_MOESM1_ESM.pdf]

Supplementary Figures and Legends

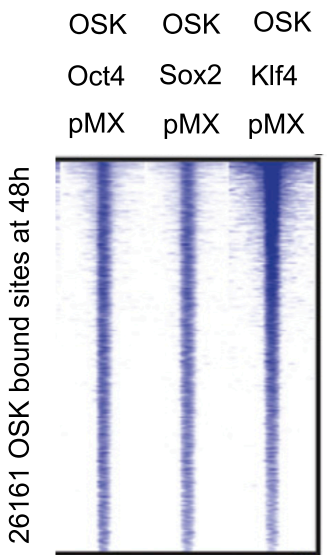

Supp Fig1. Similarities between individual retroviral based and poly-cistronic based system. A. Heatmap of ChIP-Seq signal for Oct4, Sox2 and Klf4 using pMX (individual retroviral), for sites co-bound by OSK (polycistronic) at 48 hr of OSKM-induced reprogramming. The blue color represents ChIP-Seq signal. Each row represents an OSK co-bound peak.

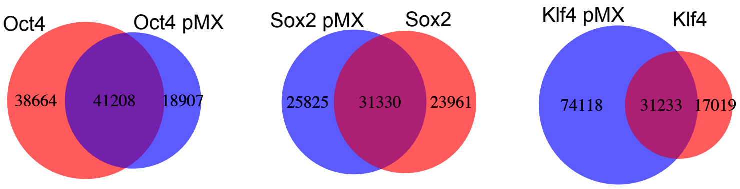

Supp Fig2. Venn diagram of mouse individual retroviral based (pMX) OSK peaks and polycistronic based OSK peaks. The numbers in the circle indicates the number of peaks. The overlapped number of peaks are statistical significant (p-value < 10^{-10}, hypergeometric test)

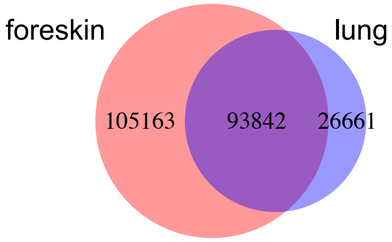

Supp Fig3. Venn diagram of DNaseI hypersensitive sites (broad peaks) between human foreskin newborn fibroblasts and human lung fetal fibroblasts. The numbers in the circle indicates the number of peaks.

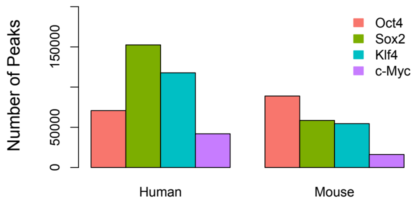

Supp Fig4. Bar plot of the number of the identified OSKM ChIP-Seq peaks in human and mouse

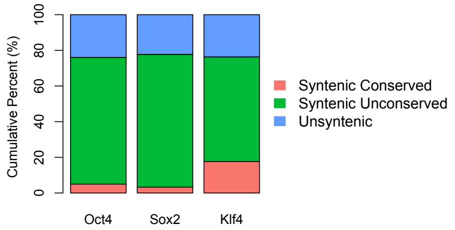

Supp Fig5. Distribution of mouse individual retroviral based (pMX) syntenic conserved, syntenic unconserved and unsyntenic peaks for Oct4, Sox2 and Klf4.

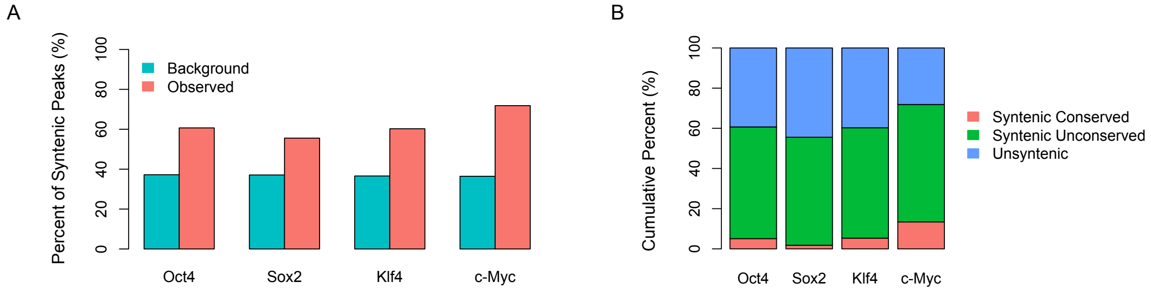

Supp Fig6. Map human OSKM peaks to mouse show limited conservation. A. Percentage of human OSKM peaks that can be mapped to mouse. B. Distribution of human syntenic conserved, syntenic unconserved and unsyntenic peaks for each factor.

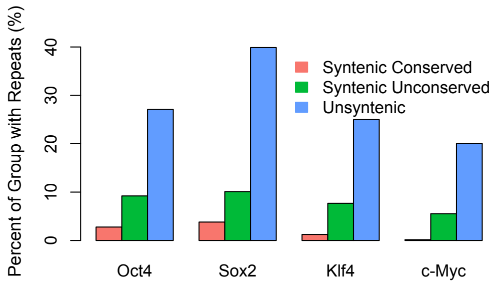

Supp Fig7. Percentage of the human syntenic conserved, syntenic unconserved and unsyntenic group of peaks that contain human repeat sequences

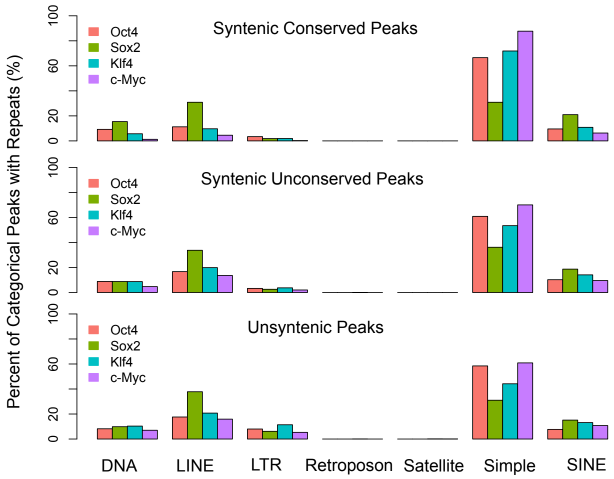

Supp Fig8. Percentages of human peaks that contain specific type of mouse repeat sequences. Seven major types of repeat, i.e. DNA (DNA transposon elements), LINE (Long interspersed nuclear elements), LTR (Long terminal repeats), Retroposon (Transposons via RNA intermediates), Satellite (Satellite DNA which belongs to tandem repeats), Simple (Simple repeats) and SINE (Short interspersed nuclear elements) are calculated.

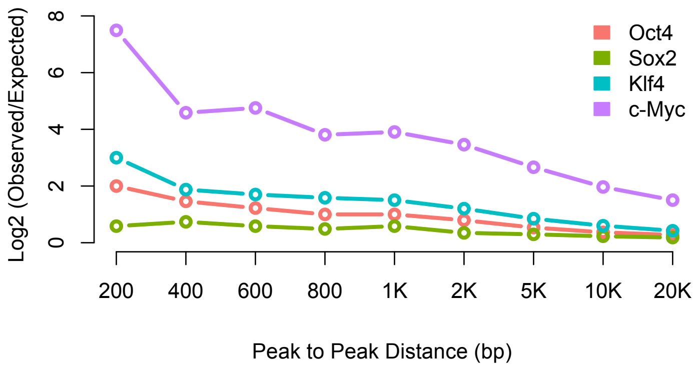

Supp Fig9. Log2 fold enrichment of distances between human syntenic peaks in mouse and mouse peaks compared to random background.

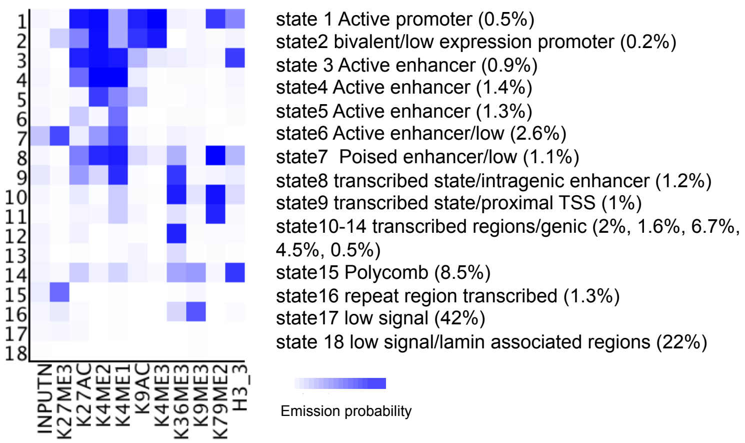

Supp Fig10. 18 chromatin state model for mouse 48 hours post induction of OSKM based on nine histone marks. This figure is taken from Chronis et al. The value in the heatmap represents the enrichment of that histone mark in that learned chromatin state. The value in the brackets represents the percentage of genome that is occupied by that specific chromatin state.

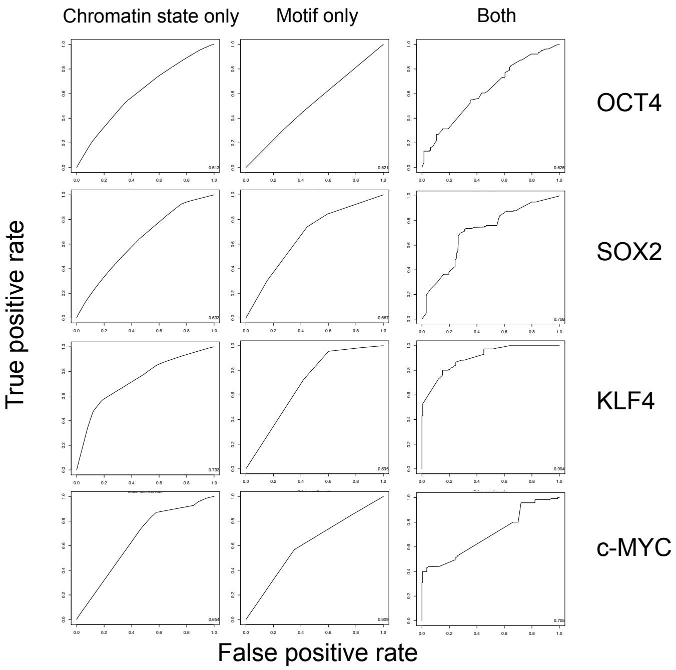

Supp Fig11. ROC Curve of Bayesian classifier for classifying peaks into conserved or unconserved with chromatin state only, motif only or both information in the model.
